# Sexual behavioural risk, partnership contexts, and correlates of condomless sexual intercourse among men newly diagnosed with HIV in Shanghai, China

**DOI:** 10.1371/journal.pgph.0007258

**Published:** 2026-11-11

**Authors:** Min Xi, Joshua B. Mendelsohn, Yinzhong Shen, Hua Cheng, Sandra Bullock, Veronika Moravan, Ann N. Burchell, Sharmistha Mishra, Xueyun Wu, Darrell H.S. Tan, Alan Li, Mona Loutfy, Liviana Calzavara

**Affiliations:** 1 Dalla Lana School of Public Health, University of Toronto, Toronto, Ontario, Canada; 2 Toronto General Hospital Research Institute, University Health Network, Toronto, Ontario, Canada; 3 ICES, Toronto, Ontario, Canada; 4 College of Health Professions, Pace University, New York, New York, United States of America; 5 Department of Infection and Immunity, Shanghai Public Health Clinical Center, Fudan University, Shanghai, China; 6 Shanghai Municipal Centre for Disease Control and Prevention, Shanghai, China; 7 VM Stats, Toronto, Ontario, Canada; 8 MAP Centre for Urban Health Solutions, Unity Health Toronto, Toronto, Ontario, Canada; 9 Department of Family and Community Medicine, Temerty Faculty of Medicine, University of Toronto, Toronto, Ontario, Canada; 10 Institute of Health Policy, Management, and Evaluation, University of Toronto, Toronto, Ontario, Canada; 11 Division of Infectious Diseases, St Michael’s Hospital, Unity Health Toronto, Toronto, Ontario, Canada; 12 Department of Medicine, Faculty of Medicine, University of Toronto, Toronto, Ontario, Canada; 13 Institute of Medical Science, University of Toronto, Toronto, Ontario, Canada; 14 Maple Leaf Clinic, Toronto, Ontario, Canada; 15 Women’s College Hospital, Toronto, Ontario, Canada; The University of Sydney, AUSTRALIA

## Abstract

Given low HIV pre-exposure prophylaxis (PrEP) and treatment as prevention uptake, understanding patterns of condomless sexual intercourse is critical for developing HIV prevention strategies in China, where HIV disproportionately affects men through sexual transmission. We quantified condom use and identified correlates of condomless sexual intercourse among men in Shanghai, China. We recruited 151 cisgender men ≥ 18 years within 60 days of their HIV diagnosis from Shanghai’s sole HIV treatment center (05/2021-03/2022). Participants were surveyed about their sexual behavioural risks within six months before survey completion. Condom use was categorized into mutually exclusive groups: low-risk (always used condoms), moderate-risk (condomless with one primary partner only), or high-risk (condomless with ≥1 non-primary partner or >1 primary partner). We used sequential Firth logistic regression to identify correlates of condomless sexual intercourse. Of 151 participants, 4.6% ever used HIV PrEP; at survey completion, 39.7% initiated antiretroviral therapy (ART) and 1.3% achieved viral suppression. Of 129 participants who engaged in sexual intercourse within six months before survey completion, 69.5% only had sex with men and 55.6% reported concurrent sex partners. One-fifth (21.7%) always used condoms (range: male partners: 11.6%-25.9%; female partners: 0%-50%). Among the 129 participants, older age (aOR per 5-year increase = 1.68;95%CI:1.20,2.51;p = 0.0018), lifetime sexually transmitted infections (aOR=3.17;95%CI:1.08-10.02;p = 0.0357), and multiple sex partners (2–3 partners: aOR=10.3;95%CI:3.45-37.48;p < 0.0001; ≥ 4 partners: aOR=17.17;95%CI:5.08-71.94;p < 0.0001) were associated with increased odds of high-risk intercourse (compared with low-/moderate-risk). In contrast, restricting to participants reporting low-/moderate-risk intercourse, ≥ 4 partners (aOR=0.10;95%CI:0.01-0.48;p = 0.0112) reduced the odds of moderate-risk intercourse. Overall, condomless sexual intercourse was common among men newly diagnosed with HIV in Shanghai, who often had multiple and concurrent partners. These findings, alongside suboptimal PrEP uptake, ART initiation, and viral suppression, underscore the need for comprehensive HIV prevention strategies integrating drivers of concurrent partnerships, publicly funded condom distribution, expanded PrEP use, and timely ART initiation among men with large sexual networks.

## Background

In 2022, 1.23 million people were living with HIV in China [1]. In China, HIV disproportionately affects men [2]. Between 1994 and 2019, men experienced two- to four-fold higher age-standardized HIV incidence rate compared to women [3]. Notably, men who have sex with men in China have experienced sustained increases in HIV prevalence from 2003 to 2018 [4,5]. Chinese national surveillance data indicated that sexual transmission has become the primary mode of HIV transmission [6]. From 2009 to 2020, the proportion of new HIV acquisitions attributed to heterosexual transmission increased from 48.3% to 74.2%, while infections attributed to same-sex transmission increased from 9.1% to 23.3% [6]. These trends underscore the growing importance of understanding sexual transmission dynamics among Chinese men to inform HIV prevention strategies for this population.

HIV acquisition among Chinese men is shaped by multiple sex partners, diverse partnership contexts (e.g., men or women primary, casual, or sex worker partners), and sociocultural pressures [7–9]. Sociocultural pressures surrounding heterosexual relationships, marriage, and fatherhood may lead some men who have sex with men to engage in heterosexual relationships while maintaining sexual relationships with other men, creating overlapping sexual networks that may facilitate HIV transmission [10–12]. National reports indicated that the proportion of men who have sex with men reporting heterosexual marriage increased from 19.3% in 2008 to 27.9% in 2011, illustrating the complexity of sex partner patterns among Chinese men [9].

Although biomedical HIV prevention strategies such as immediate antiretroviral therapy (ART) initiation [13] and pre-exposure prophylaxis (PrEP) [14] were introduced in China in 2016 and 2020, respectively, uptake remains limited. In 2022, less than 50% of men who have sex with men in Shanghai and Chengdu initiated ART [15]. In 2026, despite 82.0% of men who have sex with men being aware of PrEP, only 8.8% had ever used it, with high cost identified as a key barrier to uptake [16]. Additionally, HIV PrEP does not prevent other sexually transmitted infections (STI). In this context, condoms remain an important prevention strategy for HIV and other STI [6]. However, inconsistent condom use and condomless sexual intercourse remain common among Chinese men [17–19], with prevalence ranging from 17% [20] to 66% [17,18] across partnership contexts and sexual acts (e.g., vaginal or anal intercourse). Among Chinese men who have sex with men, condomless sexual intercourse has been associated with rural residence [18], drug or alcohol use [17,21], and lower income [22]. Condomless vaginal intercourse has been more frequently reported among older, married, and less educated straight men [10].

Despite this growing body of literature, important knowledge gaps remain. Previous studies of condomless sexual intercourse among Chinese men have primarily focused on specific sexual contexts (e.g., casual or transactional sex [23], men involved with multiple sex partners [20]) with assessment of condom use typically limited to the participant’s most recent sexual encounter [24]. To date, the full range of partnership contexts and sexual acts in which Chinese men typically engage have not been comprehensively characterized. Moreover, there is limited evidence on how key behavioural risks and clinical factors, such as the number of sex partners, number of sexual intercourse events, history of STI, and HIV testing influence condom use among Chinese men. A more detailed understanding of sex partners and condom use among Chinese men is critical for characterizing HIV risk and refining HIV prevention and counselling interventions.

To address these knowledge gaps, we used an individual-level survey to describe sexual behavioural risk, partnership context, and condom use among adult men newly diagnosed with HIV in Shanghai, China, recruited between May 11, 2021, and March 7, 2022, and to identify sociodemographic and sexual behavioural risk correlates of different levels of condom use.

## Methods

### Ethics statement

This study received ethics approval from the University of Toronto Research Ethics Board (Protocol #35006) and the SPHCC Ethics Committee (Protocol #2019-S033-02). Written informed consent was obtained from all participants.

### Study setting

Our study was conducted at the downtown satellite clinic of the Shanghai Public Health Clinical Center (SPHCC) in Hongkou District, Shanghai, the only designated hospital for HIV ART in Shanghai. Eligible participants, recruited from the SPHCC from May 11, 2021, to March 7, 2022, completed an interviewer-administered cross-sectional survey and were followed for nine months (until December 7, 2022) via chart review to collect clinical information (e.g., viral load, viral suppression [25]). This paper focusses on cross-sectional survey findings.

### Participants and recruitment

Following check-in, new clients were approached sequentially by a trained interviewer in the waiting room. Clients in the waiting room were either people living with HIV or accompanying someone receiving care. To maintain privacy, clients were asked if they were willing to move to a nearby alcove to receive study information and complete eligibility screening. Clients were eligible to participate if they: (1) were men (male sex at birth or transgender men) ≥18 years, (2) were residing in Shanghai, (3) spoke and comprehended Mandarin enough to be able to understand the consent form and survey, and (4) were within 60 days of their confirmatory HIV diagnosis in Shanghai. We excluded clients who were incapable of giving informed consent, including those who required a guardian or other third-party to make healthcare decisions.

### Data collection

Participants could complete the baseline survey immediately after written informed consent or at a later visit; all participants completed the survey within one week of screening. Survey responses were captured using pen and paper by a trained interviewer in a private room and double-entered into Epidata EntryClient 4.6, (EpiData Association, Denmark, https://epidata.dk/) by study staff.

### Measures

The questionnaire collected information on socio-demographics, STI and HIV testing history, history of ART and HIV PrEP use, and sexual behavioural risk based on self-report. Socio-demographics included age, marital status (currently married or cohabiting with a partner; other), education (high school or less; vocational school/college; professional, undergraduate, or graduate degree), income, Shanghai residency status (permanent; temporary), and sexual identity (gay; straight; straight but has sex with men; bisexual/other).

Participants reported any lifetime STI diagnoses (gonorrhea, syphilis, chlamydia, genital herpes, or other STI diagnosed by a healthcare provider) and were categorized as ever having had an STI in their lifetime (yes/no). Participants reported HIV PrEP use prior to positive HIV test and ART use following diagnosis. HIV testing history included total number of lifetime HIV tests, year of first HIV test, lifetime HIV self-test use (yes/no), and whether participants tested positive on their first HIV test. The average number of HIV tests per year was derived by dividing the total number of lifetime HIV tests by the number of years since the participant’s first HIV test. Men were then categorized as: (1) <1 year of test history, (2) <1 test per year, (3) 1 to <2 tests per year, (4) ≥2 tests per year, or (5) missing/don’t know.

Sexual behavioural risk included questions on partnership context, intercourse events, and condom use. Participants reported whether they had ever had sexual activity with men only, women only, or with men and women. Participants reported their sexual activity with seven partnership contexts: (1) primary male partners, (2) primary female partners, (3) casual male partners, (4) casual female partners, (5) male sex work partners, (6) female sex work partners, and (7) male sex work client partners. Primary partners were defined as “a sex partner with whom you have an ongoing sexual and emotional relationship; this partner is the partner with whom you feel most intimate.” Casual sex partners were defined as “non-primary partners who you have neither paid nor been paid for sexual activity.” Sex work partners were defined as people “who you paid money or gave something of value in exchange for sex.” Sex work clients were defined as people “who paid you money or gave you something of value in exchange for sex.” Based on these responses, we determined whether participants were involved in concurrent sexual partnerships over the past six months. Men self-reported the number of partners by partnership context, sex acts with each partner (receptive, insertive, both or neither with male partners or vaginal, anal, both or neither with female partners), and condom use frequency during the most recent ten intercourse events for each partnership context. These data were combined to classify participants into four mutually exclusive risk-based (based on partnership context and condom use) categories (Fig 1): (1) no sexual intercourse partners (no risk), (2) always use condoms with every partner (low-risk intercourse), (3) condomless intercourse with only one primary partner and always used condoms during intercourse with other partners (moderate-risk intercourse), or (4) condomless intercourse with someone other than just one primary partner (high-risk intercourse). Participants who only reported condomless sex with one primary partner were retained as a separate risk level for analysis (moderate-risk intercourse), as having intercourse with one’s primary partner is often perceived as being of lower risk than with other partner types due to greater trust, familiarity, intimacy, and commitment [26–31]. In contrast, condomless intercourse involving casual, multiple, or concurrent partners (high-risk intercourse) increases opportunities for HIV transmission across sexual networks and has consistently been associated with greater HIV acquisition risk [32]. Participants without a primary partner could be categorized as no risk, low-, or high-risk.

**Fig 1:**
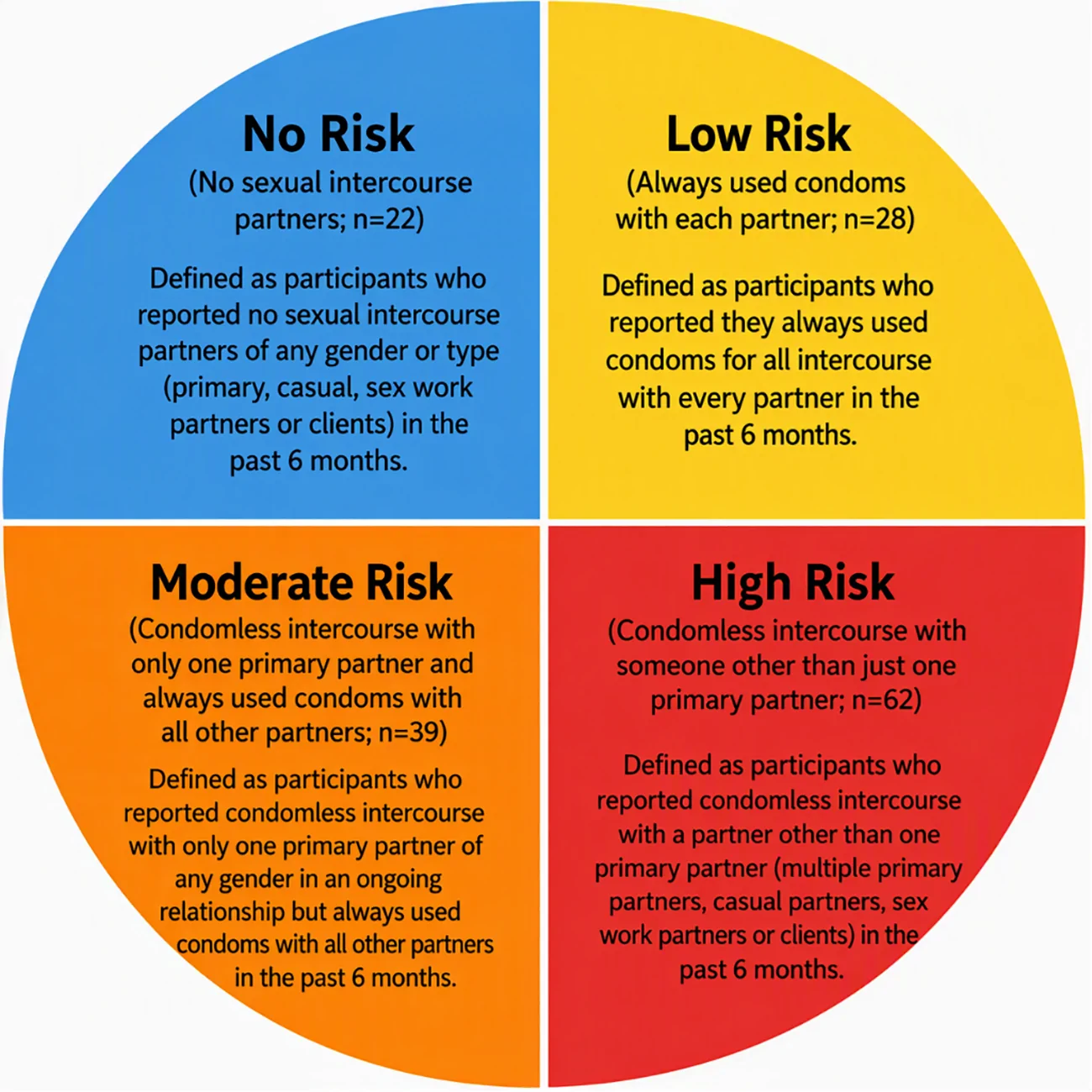
Definitions of no risk, low risk, moderate risk and high risk categories based on condom use.

### Statistical analyses

Descriptive statistics were generated for all covariates of interest. Bivariate association between the four condom use groups and covariates were tested using chi-square or Fisher’s exact test for categorical, Kruskal-Wallis test for ordinal, or ANOVA for continuous variables with Tukey’s Honestly Significant Difference post-hoc test. Firth logistic regression, the standard analytical method for handling binary outcomes in small datasets [33], was used to obtain crude (OR) and adjusted (aOR) odds ratios and 95% confidence intervals (CI) for associations between condom use and sociodemographic characteristics, sexual identity and behavioural risks, and HIV testing.

We used a sequential Firth logistic regression approach to reflect the ordered low-, moderate-, and high-risk intercourse categories and identify correlates associated with these categories while allowing associations to differ across categories. Men who did not report any sexual intercourse partners were not included. In Step 1 of the regression, we estimated the odds of reporting high-risk versus low- and moderate-risk intercourse among all participants who had engaged in sexual intercourse in the six months prior to survey completion. Step 2 was restricted to participants who reported only low- or moderate-risk intercourse in the six months prior to survey completion and estimated the odds of reporting moderate- versus low-risk intercourse. In brief, Step 1 allowed us to isolate correlates of high-risk intercourse using our full sample while Step 2 allowed us to differentiate correlates of low- versus moderate-risk intercourse. Both steps included the same set of prespecified covariates that were selected a priori based on existing literature, including demographic characteristics, sexual behavioural risk measures, STI history, and HIV testing frequency. Highly correlated variables were not included together to minimize multicollinearity. P-values were two-sided and p < 0.05 was used to determine statistical significance. In a sensitivity analysis, we repeated the sequential Firth logistic regression among men who only engaged in sex with men to assess whether associations differed (i.e., magnitude and direction of associations) from the main analysis that included all participants. Analyses were conducted using R software version 4.4.2 (R Foundation for Statistical Computing, Vienna, Austria [34]).

## Results

### Participant sociodemographic characteristics

Of 409 clients approached, 39 (9.5%) declined to answer eligibility screening questions, 207 (50.6%) were ineligible, and 163 (39.8%) were eligible. Among the 207 who were ineligible, reasons included: diagnosed more than 60 days prior to screening (n = 195; 94.2%), did not reside in Shanghai (n = 6; 2.9%), refused to answer the age or gender question (n = 3; 1.4%), could not communicate in Mandarin (n = 1; 0.5%), or was already enrolled in the study (n = 1; 0.5%). Of the 163 who were eligible, 12 (7.4%) did not enroll or did not complete the study processes, leaving 151 study participants. Although transgender men were eligible for the study, no men indicated that they were transgender upon screening.

Of 151 participants, 22 (14.6%) reported no sexual intercourse in the six months prior to survey completion. Of the remaining 129 participants who reported intercourse in the six months prior to survey completion, 28 (18.5%) reported low-risk intercourse (always used condoms), 39 (25.8%) reported moderate-risk intercourse (condomless sexual intercourse with one primary partner only), and 62 (41.1%) reported high-risk intercourse (condomless sexual intercourse with ≥1 non-primary partner or >1 primary partner; Table 1). Mean age at survey completion was 32.1 (standard deviation [SD]: 11.3); participants who reported no sexual intercourse (41.0; SD: 16.7) were older compared to participants who reported low- (28.5; SD: 6.1), moderate- (29.1; SD: 7.1), or high-risk intercourse (32.6; SD: 11.5; p = 0.0002). Most participants had vocational/college education (n = 92; 60.9%), were not married (n = 122; 80.8%), and self-identified as gay (n = 31; 62.3%) or bisexual (n = 16; 20.5%) at the time of survey completion. Approximately half were permanent Shanghai residents (n = 71; 47.0%) versus temporary residents (n = 80; 53.0%). Apart from age, sociodemographic characteristics did not differ significantly across condom use categories.

**Table 1:** Participant sociodemographic characteristics by condom use in the six months prior to survey completion (N = 151).

Characteristic	Total n (%)	No risk: No sexual intercourse partners n (%)	Low risk: Always use condoms with each partner n (%)	Moderate risk: Condomless intercourse with only one primary partner n (%)	High risk: Condomless intercourse with multiple partners n (%)	p-value
**Total**	151 (100)	22 (14.6)	28 (18.5)	39 (25.8)	62 (41.1)	
**Age at interview**						
Mean (SD)	32.1 (11.3)	41 (16.7)	28.5 (6.1)	29.1 (7.1)	32.6 (11.5)	**F = 7.16** **df = 3, 147** **p=0.0002^a^**
Range	18-71	23-71	18-43	19-44	19-66	
**Education**						
≤ High school	33 (21.9)	8 (24.2)	4 (12.1)	8 (24.2)	13 (39.4)	0.6762^b^
Vocational school/college	92 (60.9)	10 (10.9)	19 (20.7)	25 (27.2)	38 (41.3)	
Professional/undergraduate/graduate degree	26 (17.2)	4 (15.4)	5 (19.2)	6 (23.1)	11 (42.3)	
**Income (in 1000's)** ^**c**^						
Median (IQR)	10.0 (6.5, 15.0)	10.0 (6.9, 15.0)	10.0 (7.0, 18.1)	10.0 (5.0, 15.0)	10.0 (7.0, 15.0)	0.7350^d^
**Marital Status**						
Currently married	29 (19.2)	6 (20.7)	5 (17.2)	7 (24.1)	11 (37.9)	0.7788^b^
Not currently married	122 (80.8)	16 (13.1)	23 (18.9)	32 (26.2)	51 (41.8)	
**Shanghai residence status**						
Permanent	71 (47.0)	14 (19.7)	15 (21.1)	16 (22.5)	26 (36.6)	0.2541^b^
Temporary	80 (53.0)	8 (10.0)	13 (16.2)	23 (28.7)	36 (45.0)	
**Sexual identity**						
Gay	94 (62.3)	11 (11.7)	17 (18.1)	26 (27.7)	40 (42.6)	0.5001^b^
Straight	16 (10.6)	6 (37.5)	2 (12.5)	3 (18.8)	5 (31.2)	
Heterosexual, but has sex with men	6 (4.0)	1 (16.7)	1 (16.7)	2 (33.3)	2 (33.3)	
Bisexual	31 (20.5)	3 (9.7)	8 (25.8)	7 (22.6)	13 (41.9)	
Other/don’t know/missing	4 (2.6)	1 (25.0)	0 (0)	1 (25.0)	2 (50.0)	

^a^Analysis of variance test (ANOVA). Statistically significant differences in Tukey Honestly Significant Difference post-hoc testing: No sex partners versus always use condoms with each partner: p = 0.0004; No sex partners versus condomless intercourse with only one primary partner: p = 0.0003; No sex partners versus condomless intercourse with multiple partners: p = 0.0106.

^b^Fisher’s exact test.

^c^n = 151, 3 missing were imputed with median.

^d^Kruskall-Wallis test.

Abbreviations: n = number of participants; SD = standard deviation; IQR = interquartile range.

Median time from first positive HIV test to survey completion was 36 days (interquartile range [IQR]: 20.5, 56.0) and did not differ between condom use groups (p = 0.2848). Only seven (4.6%) participants reported taking PrEP before their HIV diagnosis. One-third (n = 60; 39.7%) of participants had initiated ART 0–67 days prior to survey completion and only two (1.3%) achieved viral suppression at the time of survey completion.

### Partnership context

Most participants only had sex with men (n = 105; 69.5%) in the six months prior to survey completion; few had sex with both men and women (n = 12, 7.9%) or only women (n = 12, 7.9%), with no statistically significant differences across condom use categories, for those reporting sex (p = 0.6807; Table 2). Participants reported a median of two (IQR: 1–3) sex partners in the six months prior to survey completion. Half (n = 84; 55.6%) reported concurrent sex partners during this time; all participants who had sex with women reported concurrent sex partners. Most participants reported one (n = 82; 54.3%) or two (n = 44; 29.1%) different partnership contexts (female or male primary, casual, sex worker partners) in the six months prior to survey completion. The most common partnership contexts were casual male partners (n = 81; 53.6%), primary male partners (n = 67; 44.4%), and primary female partners (n = 16; 10.6%). Few reported male sex worker partners (n = 6; 4.0%), casual female partners (n = 5; 3.3%), or female sex worker partners (n = 4; 2.6%). No participants reported having male sex work client partners.

**Table 2:** Participant sexual and HIV testing behaviours by condom use in the six months prior to survey completion (N = 151).

Characteristic	Total n (%)	No risk: No sexual intercourse partners n (%)	Low risk: Always use condoms with each partner n (%)	Moderate risk: Condomless intercourse with only one primary partner n (%)	High risk: Condomless intercourse with multiple partners n (%)	p-value
**Total**	151 (100)	22 (14.6)	28 (18.5)	39 (25.8)	62 (41.1)	
**Lifetime, had consensual sex with…**						
Men only	91 (60.3)	12 (13.2)	17 (18.7)	26 (28.6)	36 (39.6)	0.2962^a^
Women only	16 (10.6)	6 (37.5)	2 (12.5)	3 (18.8)	5 (31.2)	
Both men and women	44 (29.1)	4 (9.1)	9 (20.5)	10 (22.7)	21 (47.7)	
**Sexual activity in the last 6 months with…**						
Men only	105 (69.5)	0 (0)	24 (22.9)	32 (30.5)	49 (46.7)	0.6807^b^
Women only	12 (7.9)	0 (0)	2 (16.7)	5 (41.7)	5 (41.7)	
Both	12 (7.9)	0 (0)	2 (16.7)	2 (16.7)	8 (66.7)	
Neither	22 (14.6)	22 (100)	0 (0)	0 (0)	0 (0)	
**Sexual activity in the last 6 months with…**						
Men or both men & women	117 (77.5)	0 (0)	26 (22.2)	34 (29.1)	57 (48.7)	0.7250^b^
Women only	12 (7.9)	0 (0)	2 (16.7)	5 (41.7)	5 (41.7)	
Neither	22 (14.6)	22 (100)	0 (0)	0 (0)	0 (0)	
**Number of partnership contexts**						
0 – no intercourse	22 (14.6)	22 (100)	0 (0)	0 (0)	0 (0)	0.2295^b^
1	82 (54.3)	0 (0)	19 (23.2)	29 (35.4)	34 (41.5)	
2	44 (29.1)	0 (0)	9 (20.5)	10 (22.7)	25 (56.8)	
3	3 (2.0)	0 (0)	0 (0)	0 (0)	3 (100)	
**Number of partners**						
N	149	22	28	38	61	**<0.0001** ^**c**^
Mean (SD)	2.4 (2.3)	0 (0)	2.5 (1.5)	1.5 (1.0)	3.8 (2.7)	
Median (IQR)	2 (1, 3)	0 (0, 0)	2 (1, 4)	1 (1, 1.8)	3 (2, 5)	
Range	0–15	0, 0	1, 5	1, 4	1, 15	
**Concurrent partners (6 months)**						
No (incl. no sex)	67 (44.4)	22 (32.8)	10 (14.9)	28 (41.8)	7 (10.4)	**<0.0001** ^**b**^
Yes	84 (55.6)	0 (0)	18 (21.4)	11 (13.1)	55 (65.5)	
**Concurrent male and female partners (6 months)**						
No (incl. no sex)	139 (92.1)	22 (15.8)	26 (18.7)	37 (26.6)	54 (38.8)	0.4578^b^
Yes	12 (7.9)	0 (0)	2 (16.7)	2 (16.7)	8 (66.7)	
**Number of HIV tests taken**						
Median (IQR)	2.0 (1.0, 6.0)	1.0 (1.0, 2.8)	3.5 (2.0, 5.2)	2.0 (1.0, 5.5)	3.0 (2.0, 10.0)	**0.0030** ^**d**^
Don’t know/missing *[n (%)]*	1 (0.7)	0 (0)	0 (0)	0 (0)	1 (100)	
**Use of home (self) HIV test**						
No	53 (35.1)	15 (28.3)	7 (13.2)	12 (22.6)	19 (35.8)	**0.0083** ^**a**^
Yes	96 (63.6)	7 (7.3)	21 (21.9)	27 (28.1)	41 (42.7)	
Don’t know/missing	2 (1.3)	0 (0)	0 (0)	0 (0)	2 (100)	
**HIV positive at first HIV test** ^**e**^						
No	91 (60.3)	8 (8.8)	18 (19.8)	21 (23.1)	44 (48.4)	**0.0218** ^**a**^
Yes	59 (39.1)	14 (23.7)	10 (16.9)	18 (30.5)	17 (28.8)	
Don’t know/missing	1 (0.7)	0 (0)	0 (0)	0 (0)	1 (100)	
**Average Number of HIV tests per year since testing start**						
<1 year test history	68 (45)	14 (20.6)	12 (17.6)	20 (29.4)	22 (32.4)	**0.0257** ^**a**^
<1 test per year	26 (17.2)	7 (26.9)	4 (15.4)	6 (23.1)	9 (34.6)	
1-1.9 tests per year	18 (11.9)	1 (5.6)	5 (27.8)	3 (16.7)	9 (50.0)	
≥2 tests per year	38 (25.2)	0 (0)	7 (18.4)	10 (26.3)	21 (55.3)	
Don’t know/missing	1 (0.7)	0 (0)	0 (0)	0 (0)	1 (100)	

^a^Fisher’s exact test.

^b^Fisher’s exact test (excluding n = 22 men with no sex partners).

^c^Kruskal-Wallis test (excluding n = 22 men with no sex partners). Statistically significant differences in Dunn’s post-hoc testing: Always use condoms versus condomless intercourse with only one primary partner: p = 0.013; Always use condoms versus condomless intercourse with multiple partners: p = 0.013; Condomless intercourse with only one primary partner versus condomless intercourse with multiple partners: p < 0.0001.

^d^Kruskal-Wallis test. Statistically significant differences in Dunn’s post-hoc testing: No sex partners versus always use condoms with each partner: p = 0.0197; No sex partners versus condomless intercourse with multiple partners: p = 0.0028.

^e^HIV positive at first HIV test includes first HIV tests that was either positive or indeterminate.

Abbreviations: n=number of participants; IQR=interquartile range.

### Participant HIV testing behaviour

The median lifetime number of HIV tests was two (IQR: 1, 6; Table 2). Of 38 (25.2%) participants who tested ≥2 times per year on average, half (n = 21; 55.3%) reported high-risk intercourse, one-quarter (n = 10; 26.3%) reported moderate-risk intercourse, one-fifth (n = 7; 18.4%) reported low-risk intercourse, and none reported no sexual intercourse in the six months prior to survey completion(p = 0.0252). Of 96 (63.6%) participants who reported using an HIV self-test, 41 (42.7%) reported high-risk intercourse, 27 (n = 28.1%) reported moderate-risk intercourse, 21 (21.9%) reported low-risk intercourse, and 7 (7.3%) reported no sexual intercourse partners (p = 0.0083). Of 59 (39.1%) participants who reported testing positive at their first HIV test, 17 (28.8%) reported high-risk intercourse, 18 (n = 30.5%) reported moderate-risk intercourse, 10 (16.9%) reported low-risk intercourse, and 14 (23.7%) reported no sexual intercourse partners (p = 0.0218).

### Condom use

Of 129 participants who engaged in sexual intercourse, 28 (21.7%) always used condoms and 101 (78.3%) reported inconsistent use of condoms or never using condoms during sexual intercourse. Among 67 participants who reported having a primary male partner, only 11 (16.4%) reported always using a condom (Fig 2). Among 81 participants who reported having a casual male partner, 21 (25.9%) reported always using a condom. Among six participants who reported having a partner who was a male sex worker, only one (16.7%) reported always using a condom. Consistent condom use did not differ by type of sexual act (receptive, insertive, neither, or both) across male partnership contexts (p > 0.05).

**Fig 2:**
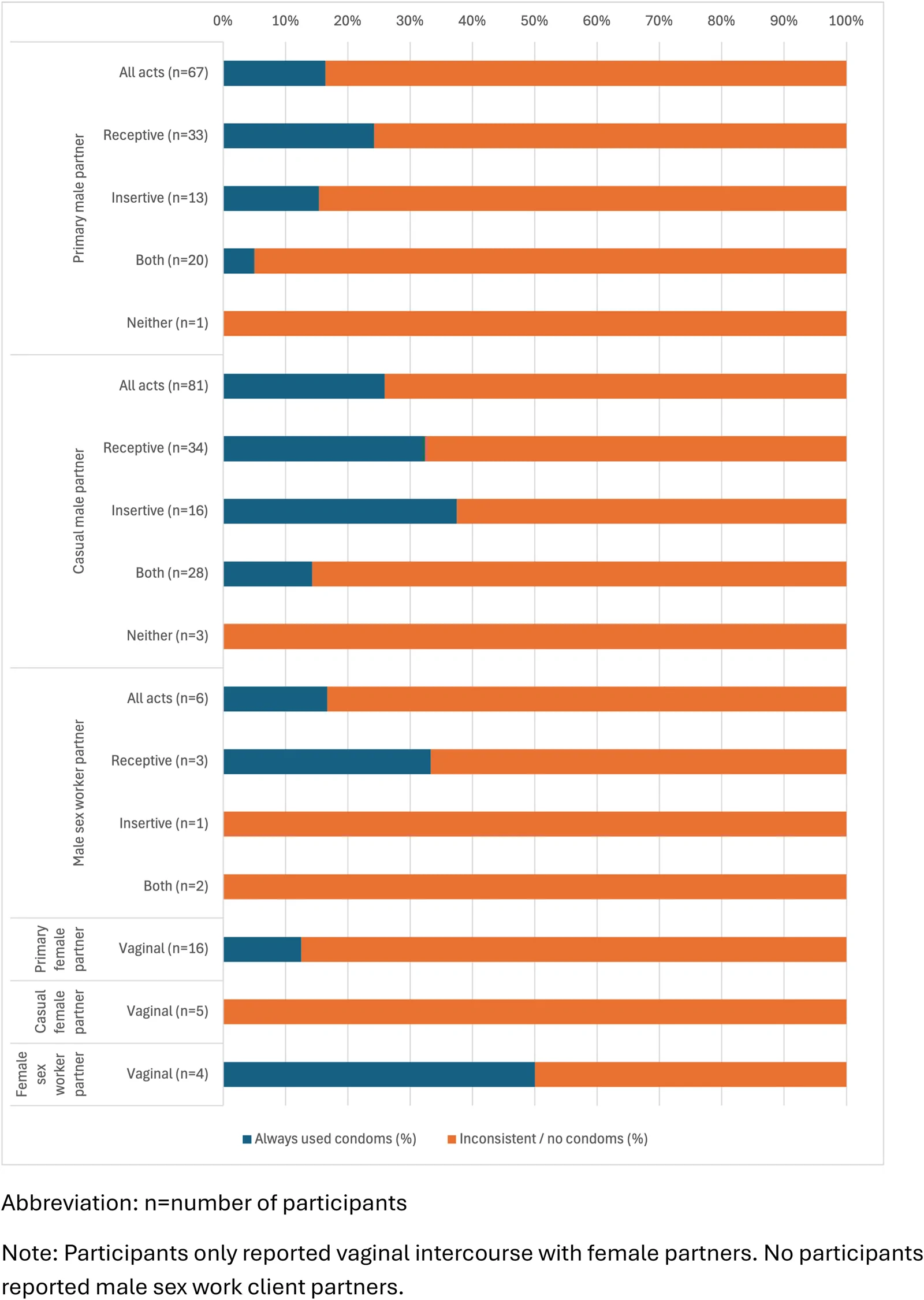
Condom use among adult men newly diagnosed with HIV in Shanghai, China, by sex partner and intercourse type in the six months prior to survey completion (N = 129).

All participants who reported having a female partner only reported vaginal intercourse; no anal intercourse was reported. Among 16 participants who reported having a primary female partner, only two (12.5%) reported always using a condom (Fig 2). Always using condoms was reported by none of the five participants who reported having a casual female partner and by 50% of the four participants who reported having a female sex worker partner.

### Predictors of high-risk intercourse

Table 3 shows the unadjusted and adjusted odds of high-risk intercourse (i.e., condomless intercourse with more than one primary partner or with any non-primary partner) versus either low- or moderate-risk intercourse (always used condoms or had condomless sexual intercourse with only one primary partner) from Step 1 of the regression model. In adjusted analyses, each five-year increase in age was associated with higher odds of high-risk intercourse (aOR: 1.68; 95% CI: 1.20, 2.51; p = 0.0018). In adjusted analyses, participants who had ever contracted a STI in their lifetime had 3.17 (95% CI: 1.08, 10.02; p = 0.0357) times higher odds of engaging in high-risk intercourse, respectively. Compared to participants who reported one partner, participants who reported 2–3 partners (aOR: 10.3; 95% CI: 3.45, 37.48; p < 0.0001) or ≥4 partners (aOR: 17.17; 95% CI: 5.08, 71.94; p < 0.0001) experienced higher odds of high-risk intercourse.

**Table 3:** Unadjusted and adjusted odds of high-risk^a^ versus low-/moderate-risk^b^ intercourse among adult men newly diagnosed with HIV in Shanghai, China, who engaged in sexual intercourse in the six months prior to survey completion.

	Unadjusted (N = 129)					Adjusted (N = 125)				
	n/N (high-risk %)	OR	95% CI	Category p-value	Covariate p-value	n/N (high-risk %)	aOR	95% CI	Category p-value	Covariate p-value
**Age at interview** *(centered, scaled by 5 years)*	–	1.24	(1.03, 1.53)		**0.0245**	–	1.68	(1.20, 2.51)		**0.0018**
**Education**					0.8680					0.8020
≤ High school [reference]	13/25 (52.0)	1.00				13/24 (54.2)	1.00			
Vocational school/college	38/82 (46.3)	0.80	(0.33, 1.94)	0.6219		35/79 (44.3)	1.40	(0.38, 5.37)	0.6160	
Professional/undergraduate/graduate degree	11/22 (50.0)	0.93	(0.30, 2.86)	0.8931		11/22 (50.0)	1.67	(0.37, 8.18)	0.5080	
**Income** *(centered, in 1000's; missing [n = 3] imputed with median)*	–	1.00	(0.99, 1.01)		0.7931	–	0.99	(0.98, 1.01)		0.2933
**Marital Status**					0.9849					0.2265
Currently married [reference]	11/23 (47.8)	1.00				11/22 (50.0)	1.00			
Not currently married	51/106 (48.1)	1.01	(0.41, 2.48)			48/103 (46.6)	2.41	(0.59, 11.09)		
**Shanghai residence status**					0.6237					0.4637
Permanent [reference]	26/57 (45.6)	1.00				23/54 (42.6)	1.00			
Temporary	36/72 (50.0)	1.19	(0.60, 2.38)			36/71 (50.7)	1.42	(0.55, 3.68)		
**Sexual identity**					0.9871					0.6272
Gay [reference]	40/83 (48.2)	1.00				38/81 (46.9)	1.00			
Straight	5/10 (50.0)	1.07	(0.3, 3.89)	0.9113		5/9 (55.6)	2.19	(0.3, 21.03)	0.4476	
Other (bisexual, heterosexual but has sex with men, other, don’t know)	17/36 (47.2)	0.96	(0.44, 2.09)	0.9259		16/35 (45.7)	0.82	(0.26, 2.49)	0.7230	
**Total number of intercourse events in the six months prior to survey completion**					0.9224					0.4147
1-6 events [reference]	32/66 (48.5)	1.00				30/64 (46.9)	1.00			
>6 events	30/63 (47.6)	0.97	(0.49, 1.92)			29/61 (47.5)	0.68	(0.27, 1.70)		
**Ever had an STI (lifetime)**					**0.0271**					**0.0357**
No [reference]	43/101 (42.6)	1.00				42/99 (42.4)	1.00			
Yes	18/27 (66.7)	2.62	(1.11, 6.51)			17/26 (65.4)	3.17	(1.08, 10.02)		
**Number of sex partners in the six months prior to survey completion**					**<0.0001**					**<0.0001**
One [reference]	7/45 (15.6)	1.00				6/44 (13.6)	1.00			
Two or three	30/47 (63.8)	8.90	(3.50, 25.20)	**<0.0001**		30/47 (63.8)	10.30	(3.45, 37.48)	**<0.0001**	
Four or more	24/35 (68.6)	10.90	(4.00, 33.20)	**<0.0001**		23/34 (67.6)	17.17	(5.08, 71.94)	**<0.0001**	
**Number of testing events (lifetime)** *(continuous)*	–	1.04	(0.99, 1.11)	0.1580	0.1580	–	1.05	(0.97, 1.15)	0.2506	0.2506

Abbreviations: N = number of participants; OR=odds ratio; aOR=adjusted odds ratio; CI = confidence interval; STI = sexually transmitted infection.

^a^High-risk intercourse refers to condomless intercourse with someone other than just one primary partner.

^b^Low-risk intercourse refers to consistent condom use with each partner. Moderate risk intercourse refers to condomless intercourse with only one primary partner and always used condoms during intercourse with other partners.

### Predictors of moderate-risk intercourse

In Step 2 of the regression model, analyses were restricted to the 67 participants who reported low- or moderate-risk intercourse. Table 4 shows the unadjusted and adjusted odds of moderate-risk intercourse (i.e., condomless sexual intercourse with a single primary partner) versus low-risk intercourse (i.e., consistent condom use). In adjusted analyses, participants who engaged in >6 intercourse events in the six months prior to survey completion had 8.00 (95% CI: 2.36, 37.41; p = 0.0005) times higher odds of moderate-risk intercourse compared to participants who engaged in ≤6 events during this period. Compared to participants who had one partner in the six months prior to survey completion, participants who had 2–3 partners (aOR: 0.34; 95% CI: 0.07, 1.39) or ≥4 partners (aOR: 0.10; 95% CI: 0.01, 0.48) experienced reduced odds of moderate-risk intercourse (p = 0.0112).

**Table 4:** Unadjusted and adjusted odds of moderate-^a^ versus low-risk^b^ intercourse among men newly diagnosed with HIV in Shanghai, China, who engaged in low- or moderate-risk intercourse in the six months prior to survey completion.

	Unadjusted (n = 67)					Adjusted N = 66)				
	n/N (moderate-risk %)	OR	95% CI	Category p-value	Covariate p-value	n/N (moderate-risk %)	aOR	95% CI	Category p-value	Covariate p-value
**Age at interview** *(centered, scaled by 5 years)*	–	1.06	(0.74, 1.52)		0.7657	–	1.14	(0.64, 2.21)	0.6620	0.6620
**Education**					0.8183					0.9806
≤ High school [reference]	8/12 (66.7)	1.00				7/11 (63.6)	1.00			
Vocational school/college	25/44 (56.8)	0.69	(0.18, 2.43)	0.5713		25/44 (56.8)	0.87	(0.12, 4.94)	0.8729	
Professional/undergraduate/graduate degree	6/11 (54.5)	0.63	(0.12, 3.13)	0.5671		6/11 (54.5)	0.98	(0.09, 13.84)	0.9890	
**Income** *(centered, in 1000's; missing [n = 3] imputed with median)*	–	0.99	(0.95, 1.00)		0.0990	–	1.00	(0.93, 1.02)		0.7945
**Marital Status**					0.9820					0.8424
Currently married [reference]	7/12 (58.3)	1.00				6/11 (54.5)	1.00			
Not currently married	32/55 (58.2)	1.01	(0.29, 3.43)			32/55 (58.2)	0.80	(0.08, 8.69)		
**Shanghai residence status**					0.3157					0.4055
Permanent [reference]	16/31 (51.6)	1.00				16/31 (51.6)	1.00			
Temporary	23/36 (63.9)	1.64	(0.63, 4.34)			22/35 (62.9)	1.74	(0.47, 7.75)		
**Sexual identity**					0.8455					0.8211
Gay [reference]	26/43 (60.5)	1.00				26/43 (60.5)	1.00			
Straight	3/5 (60.0)	0.92	(0.16, 6.05)	0.9293		2/4 (50.0)	0.33	(0.01, 15.74)	0.5526	
Other (bisexual, heterosexual but has sex with men, other, don’t know)	10/19 (52.6)	0.73	(0.25, 2.14)	0.5627		10/19 (52.6)	0.73	(0.15, 3.59)	0.6893	
**Total number of intercourse events in the six months prior to survey completion**					**0.0001**					**0.0005**
1-6 events [reference]	12/34 (35.3)	1.00				12/34 (35.3)	1.00			
>6 events	27/33 (81.8)	7.62	(2.65, 24.41)			26/32 (81.2)	8.00	(2.36, 37.41)		
**Ever had an STI (lifetime)**					0.6235					0.5676
No [reference]	33/58 (56.9)	1.00				32/57 (56.1)	1.00			
Yes	6/9 (66.7)	1.41	(0.36, 6.43)			6/9 (66.7)	0.58	(0.08, 3.88)		
**Number of sex partners in the six months prior to survey completion**					**0.0073**					**0.0112**
One [reference]	28/38 (73.7)	1.00				28/38 (73.7)	1.00			
Two or three	7/17 (41.2)	0.26	(0.08, 0.84)	**0.0234**		7/17 (41.2)	0.34	(0.07, 1.39)	0.1353	
Four or more	3/11 (27.3)	0.15	(0.03, 0.60)	**0.0064**		3/11 (27.3)	0.10	(0.01, 0.48)	**0.0036**	
**Number of testing events (lifetime)** *(continuous)*	–	0.97	(0.88, 1.06)		0.4424	–	0.95	(0.81, 1.10)		0.4910

Abbreviations: N = number of participants; OR=odds ratio; aOR=adjusted odds ratio; CI = confidence interval; STI = sexually transmitted infection.

^a^Moderate risk intercourse refers to condomless intercourse with only one primary partner and always used condoms during intercourse with other partners.

^b^Low-risk intercourse refers to consistent condom use with each partner.

### Summary of 2-step regression results

In summary, older age, lifetime STI history, and a greater number of sex partners was associated with increased odds of condomless sex with >1 primary partner or any non-primary partner. Conversely, when we restricted analyses to participants who reported low- and moderate-risk intercourse, a greater number of sex partners was associated with reduced odds of condomless intercourse with a single primary partner, while a greater number of intercourse events was associated with increased odds of condomless intercourse with a single primary partner. Sensitivity analyses restricted to men who had only engaged in sex with men in the six months prior to survey completion showed similar results.

## Discussion

In this study of men recently diagnosed with HIV in Shanghai, China, we found a high prevalence of inconsistent condom use, with approximately three-quarters of participants reporting condomless or inconsistently protected sexual intercourse. Half of our participants reported concurrent partners and a third reported sexual activity across multiple partnership types including men and women primary, casual, and sex worker partners. We found that older age, lifetime history of STI, and sexual behavioural risks including number of partners and number of intercourse events, were associated with condomless sexual intercourse. Despite the availability of biomedical prevention strategies, few had ever used PrEP (4.6%) prior to HIV diagnosis or achieved viral suppression (1.3%) at the time of survey completion. Overall, our findings provide context on behavioural risks, partnership context, sexual network structure, and structural and service factors related to HIV prevention and care.

Notably, a larger proportion of men in our study inconsistently used condoms or always engaged in condomless sexual intercourse (78%) compared to previous studies of Chinese men newly diagnosed with a sexually transmitted disease (43%) [19] or HIV (62%) [35]. These differences may reflect variations in study population and sexual behavioural risk patterns. For example, 43% of Chinese men with a regular female partner reported inconsistent condom use [19] whereas higher levels of inconsistent condom use (62%) were reported by men who have sex with men [35]. Measurement differences may also contribute. By assessing condom use across participants’ last 10 intercourse encounters rather than only the most recent one [24], our study was able to capture detailed instances of condomless sexual intercourse.

Further, our findings highlight the prevalence of overlapping sex partners among men newly diagnosed with HIV in China and the association between number of sex partners and condomless sexual intercourse. Half of our participants reported concurrent partners and one-third of participants engaged in sexual intercourse with both men and women or across two or more partnership contexts including primary and casual male and female partners. Engaging in sexual intercourse with both men and women may reflect Chinese sociocultural expectations for men to engage in heterosexual relationships, marry, and become fathers to carry on the family line [7–9]. Some men who have sex with men may maintain female partners alongside male partners to navigate these social norms. Such practices inadvertently expand HIV transmission networks (e.g., overlapping and interconnected sexual networks), underscoring the need for prevention interventions that address sociocultural drivers and reach both same-sex and heterosexual partners of Chinese men.

Consistent with previous studies [22,36,37], we found that having more sex partners was associated with condomless sexual intercourse with >1 primary or any non-primary partners, reflecting greater opportunities for HIV transmission across sexual networks. In addition to interventions focused on non-primary partners such as casual, transactional, or sex worker partners, our findings highlighted the importance of addressing HIV transmission within primary partnerships. However, this may be particularly challenging in primary relationships as condom use may be shaped by relationship dynamics. For example, trust, familiarity, intimacy, and commitment, which may reduce perceived HIV risk, while making condom use discussions more difficult and increasing willingness to forgo condoms, contributing to higher rates of condomless sexual intercourse [26–31]. As a result of Chinese sociocultural expectations for men to become fathers to carry on the family line [7–9], condom use within male-female primary partnerships may be viewed as unnecessary or inconsistent with relationship commitment.

In addition to the number of sex partners, we found that older age and lifetime history of STI were associated with higher odds of condomless sexual intercourse. These associations may reflect differences in sexual health literacy, including HIV risk perception and awareness of available HIV prevention information and services. Older generations may have had less exposure to comprehensive sexual health education [38]. Adherence to traditional sociocultural norms (e.g., Confucian values [39]) and persistent stigma surrounding sexuality [38,40,41] among older men may limit open discussion of sexual health and HIV prevention. These factors may contribute to reduced engagement with HIV testing and prevention services and lower awareness or concern regarding STI risks among older compared with younger men [42,43]. Older adults may also perceive less need for pregnancy prevention [26,44], further increasing their likelihood of condomless sexual intercourse. In a previous study, participants did not increase their use of condoms following STI diagnosis, suggesting that an STI diagnosis alone was insufficient to change sexual behaviours [45]. Stigma surrounding STI disclosure may further impede condom negotiation, particularly with non-primary sex partners, reinforcing patterns of condomless sexual intercourse [46]. These patterns highlight persistent gaps in condom-based prevention and underscore the need for complementary HIV prevention strategies.

In this context, the low uptake of HIV PrEP and delays in ART initiation observed in our study were notable. Despite documented effectiveness [47,48], few participants had used PrEP prior to HIV diagnosis. Our findings were consistent with prior evidence from China showing that although 87% of men who have sex with men who were eligible for PrEP and 82% were aware of it, only 9% had ever used PrEP [16]. High cost was identified as a key barrier to PrEP uptake among Chinese men [16]. Similarly, previous studies estimated low levels of ART initiation (47%) among men who have sex with men in China [15]. The low levels of immediate ART initiation and viral suppression in our study were suggestive of missed opportunities for realizing the prevention benefits of treatment as prevention. Expanding PrEP uptake among individuals with multiple and/or concurrent partners and increasing initiation of ART immediately following HIV diagnosis to achieve viral suppression, paired with publicly funded condom programs and education/prevention strategies that integrate behavioural interventions with routine STI care, may provide complementary strategies to reduce and prevent HIV and STI transmission.

### Limitations

This study provided a nuanced description of sex partners and behavioural risks of men in Shanghai, China, and identified factors associated with condomless sexual intercourse within this population. The study had several key limitations. Participants were recruited from a single tertiary HIV treatment center at the sole HIV treatment hospital in metropolitan Shanghai and required Mandarin proficiency. Therefore, findings may overrepresent men already connected to care and underrepresent vulnerable men who were not linked to care. Our findings may not be generalizable to undiagnosed individuals, men receiving care in other settings (e.g., private clinics, clinics outside of Shanghai), vulnerable men not connected to care, or newly diagnosed men with HIV in other regions. Moreover, participants engaged in HIV care may have had greater exposure to HIV-related education and prevention messaging, which could have influenced reported condom use behaviours relative to the broader population of men. Despite survey administration by trained interviewers in private settings, self-reported sexual behavioral risks that occurred over a 6-month period prior to survey completion were subject to social desirability and recall bias which may have led to under-reporting of condomless sexual intercourse and other sexual behavioural risks (e.g., concurrent partners, number of partners, sex worker partners). While our outcome classification was based on condom use patterns rather than partner number, individuals with more partners may have had greater opportunities for condomless intercourse across different partnerships. Thus, the association between number of sexual partners and behavioural risk categories should be interpreted with caution. Our small sample size limited the precision of regression estimates and our ability to explore potentially important effect modifiers. Though we used Firth logistic regression to mitigate biases due to our small sample size, we did not have sufficient sample size (n = 34 receptive; n = 16 insertive; n = 28 both) to explore type of sex act as an effect modifier. Our survey was cross-sectional, limiting our ability to assess temporality and hence limiting the causal interpretation of our findings. In addition, information on partners’ HIV status was incomplete, with 20% to 100% of participants reporting that they did not know their partner's HIV status depending on partnership type (e.g., male or female primary, casual, or sex worker partners). Among participants who reported a partner living with HIV, we lacked temporal information on when the partner acquired HIV relative to the participant, when the participant became aware of the partner's HIV status, and whether the partner was receiving ART and/or was virally suppressed. Future studies should explore the impact of type of sex act and partner’s HIV status on condom use. Additionally, participants were interviewed within 60 days of their confirmatory HIV diagnosis. In this 60-day period, knowledge of HIV status may have influenced sexual behaviors including condom use. However, only one-third of participants had initiated ART and just two participants reported viral suppression at the time of survey completion, which suggested that the impact of this delay on sexual behavior may have been minimized. Changes in structural constraints to service access including public ART coverage, stigma related to HIV and/or men who have sex with men, and public health policies related to mobility during and following the COVID-19 pandemic may have led to changes in sexual activity and partner dynamics that were not captured in our study and should be explored in a future study. Longitudinal studies using repeated measures or real-time data collection methods (e.g., sexual activity diaries) would more reliably characterize patterns of sexual behavioural risks and condom use among Chinese men could serve as an important follow-up to document ongoing patterns of sexual behavior.

## Conclusion

Among men newly diagnosed with HIV in Shanghai, China, condomless sexual intercourse in the previous 6 months was prevalent, particularly with primary partners and across overlapping sexual networks involving both male and female partners. Older age, prior STI diagnosis, and multiple sex partners were associated with condomless sexual intercourse. PrEP uptake, immediate ART initiation, and subsequent achievement of viral suppression among Chinese men were low. Notably, these findings revealed opportunities to reduce HIV transmission by substantially increasing PrEP access and uptake and accelerating initiation of immediate ART and supportive services targeted at reducing time to viral suppression. To accomplish these aims, there is a need for comprehensive, complementary, and free or low-cost HIV prevention interventions that address sociocultural pressures and reduce access barriers. New initiatives should include educational interventions that support STI prevention and care, expansion of access to PrEP, and enhanced support for rapid uptake of immediate ART.
